# Application of Response Surface Methodology for Modeling and Optimization of the Top Surface Ironing Process in Parts Produced by MEX 3D Printing

**DOI:** 10.3390/ma18225248

**Published:** 2025-11-20

**Authors:** Andrzej Matras

**Affiliations:** Department of Production Engineering, Faculty of Mechanical Engineering, Cracow University of Technology, 31-155 Cracow, Poland; andrzej.matras@pk.edu.pl; Tel.: +48-12-628-3228

**Keywords:** ironing, material extrusion, surface roughness, surface flatness, Response Surface Methodology, 3D printing, process efficiency, polylactic acid

## Abstract

**Highlights:**

**What are the main findings?**

- The process of surface ironing in 3D printing was analyzed.
- The simultaneous influence of the key parameters of the surface ironing process was analyzed.

**What is the implication of the main finding?**

- Surface ironing resulted in a fivefold reduction in the Ra and an eightfold reduction in the FLTq.
- Simultaneously increasing the extruder temperature, the speed, and the pass distance allows for higher process efficiency while maintaining high surface quality.

**Abstract:**

This study analyzes the influence of material extrusion (MEX) 3D printing and ironing parameters on parts made of polylactic acid (PLA), such as top surface roughness and flatness. Surface ironing is one of the post-processing methods. It is an interesting alternative to the most commonly used mechanical or chemical treatments. It does not require the use of any additional devices or substances; however, it can only be used on flat surfaces, requires additional time for application, and a flush may form at the edges of the ironed surface. The Response Surface Methodology (RSM) was used for modeling the analyzed processes. The presented methodology assumes a two-stage approach. First, the printing process of the top surface is optimized. Then, the ironing of the top surface is optimized. Improvement of surface roughness and flatness was adopted as the optimization criterion. The influence of extruder temperature, printing speed, and filament flow used during printing of the top surface, as well as extruder temperature, ironing speed, and distance between passes used during ironing, was examined. The significance of the influence of the analyzed parameters was determined using ANOVA and Pareto diagrams. The use of the applied research methodology and created mathematical models allows for determining the relationship between the optimal extruder temperature, extrusion flow, speed, and distance between passes during ironing while ensuring high process efficiency. The ironing resulted in a fivefold reduction in the Ra and an eightfold reduction in the FLTq parameters. A surface with Ra = 1.09 μm and FLTq = 3.4 μm was obtained.

## 1. Introduction

Thermoplastic materials are used in various fields of life and engineering. One of the most widely used methods of manufacturing from thermoplastic polymers is material extrusion (MEX). It allows for producing various parts, sensors, and useable items [1]. It is used in industries such as automotive (prototypes of parts, interior components, and covers), medical (prosthetics and anatomical models), electronics and electrical engineering (device cases), and industrial design (casting molds, prototypes, and concept models). Polymers such as polylactic acid (PLA), acrylonitrile butadiene styrene (ABS), polyethylene terephthalate (PETG), high-impact polystyrene (HIPS), polyamide (PA), polycarbonate (PC), and reinforced composite materials can be used in MEX [2,3,4,5]. Many researchers focus research on improving the effects of using MEX [6]. Modern optimization methods like Taguchi method [7,8], RSM [9,10], Machine Learning [11,12], and Full Factorial Design [13] can be effectively used for this purpose.

Obtaining smooth and precise surfaces of components manufactured using MEX is also possible through post-processing. It is mainly based on the use of additional mechanical [14,15], thermal [16], chemical [17], or laser polishing [18,19,20]. However, each of them requires additional equipment or substances. The situation is different in the case of surface ironing. Surface ironing consists of performing, immediately after printing a layer, additional movements of the extruder, aimed at smoothing and improving the geometric parameters of the layer. These movements can be performed both after the last layer and intermediate layers. As a result, with a specific speed and distance between passes, the flat surface of the extruder nozzle smooths the surface of the layer produced. In order to increase the efficiency of the process, the extruder is heated to the appropriate temperature and an additional small amount of material can be extruded. Despite the advantages of the ironing process, only a few studies focus on optimizing the parameters of this process. Butt et al. [21] analyze the effect of the applied ironing speed, ironing line spacing, and ironing flow on the roughness and hardness of elements printed using MEX from ABS and ASA. They note that the use of filament flow during ironing may in some cases cause an increase in the dimensions of elements made of ABS. Performing the ironing process also increases the hardness of the elements. However, the influence of extruder temperature and the interactions of tested parameters are still unknown. Sardinha et al. [22] iron surfaces of different dimensions. As a result of the process, the surface roughness is improved. They report the need for further research on the ironing process. However, it does not test the influence of process parameters on its results. Caputo et al. [23] analyze the influence of ironing parameters on the topography and surface roughness. They observe the effect of nozzle temperature on surface roughness. However, they do not use a two-stage approach. The same temperature and speed are used during printing and ironing stage. The authors [24] examined the influence of ironing speed, distance between passes, and flow on the surface roughness and mechanical properties of samples made of PLA. Ironing is performed on each layer. Based on the Box–Behnken Design, they select a set of parameters that allows for reducing roughness and improving the mechanical properties of the element. However, they do not test the effect of ironing temperature. Performing ironing on each layer is also time-consuming. The analysis does not reveal whether it is necessary to improve surface quality. Neuhaus et al. [25] investigate the influence of extrusion temperature, flow, speed, and distance between passes. They identify a significant influence of extruder temperature on surface roughness. However, they analyze the influence of each parameter separately, so the interactions between ironing parameters are not determined. This approach does not allow for simultaneous optimization of more than one parameter.

This study analyzes and optimizes selected parameters of the MEX and ironing processes. A two-stage approach was used. First, the print parameters for the top surface of the part were optimized. Then, the ironing of the top surface was carried out. The goal of the optimization was to improve roughness and flatness (expressed by the Ra and FLTq parameters) obtained as a result of surface ironing while ensuring high ironing process efficiency. As a result of using RSM, the simultaneous influence of extruder temperature, speed, distance between passes, and filament flow used during ironing on the roughness and flatness of the upper surface of PLA elements was characterized. The relations between the optimal extruder temperatures and other ironing parameters, enabling the production of surfaces characterized by low roughness and high flatness, were also determined. These results make a significant contribution to the development of knowledge about finishing processes in MEX technology and may constitute a reference point for further experimental work and mathematical modeling. The applied experimental research and the response surface method are the most efficient tools known for the analysis of processes for which the mathematical description of the occurring physical phenomena is very complex and impossible to describe. The presented research also responds to numerous discussions within various communities focused on 3D printing technology. To the author’s knowledge, this study is the first to characterize the interrelationships between key parameters of the ironing process.

## 2. Materials and Methods

### 2.1. Description of the Performed Research

During this study, the material extrusion (MEX) 3D printing process was analyzed. The study was conducted in two main stages. In the first stage (printing stage), the top surface printing process was optimized. The goal of this optimization was to achieve a high-quality top surface of the sample while ensuring high process efficiency. At this stage, the influence of variable extruder temperature (T), top surface printing speed (v), and filament flow (Q) on variations in the top surface roughness (Ra) and flatness (FLTq) parameters were tested. The range of variability of the analyzed parameters was determined based on a review of the literature and the filament manufacturer’s recommendations. The tested top surface printing parameter levels are summarized in Table 1.

During sample printing, constant process parameters were used. The layer height was 0.2 mm, wall thickness 2.2 mm, linear transitions on the top layer, 40% grid-shaped infill, bed temperature 60 °C, first layer printing temperature 210 °C, infill printing speed 220 mm/s, wall printing speed 60 mm/s, and axis acceleration 1200 mm/s^2^. The printing parameters used were selected based on the recommendations of the filament and printer manufacturers, as well as our own experience. They allowed for the formation of a stable part structure, providing a solid base for the printed top surface.

During the second stage (ironing stage), the top surface ironing process was analyzed. In this stage, the top surface of the sample was firstly printed using the optimal parameters determined in the printing stage. Then, immediately after printing, the top surface was ironed. The influence of the variable extruder temperature T_p_, ironing speed v_p_, filament flow Q_p_, and the distance between passes a_p_ used in the ironing process were tested. The range of variability of the analyzed parameters was determined based on a review of the literature, preliminary experimental studies, and the filament manufacturer’s recommendations. The tested top surface ironing parameter levels are summarized in Table 2.

**Figure 1:**
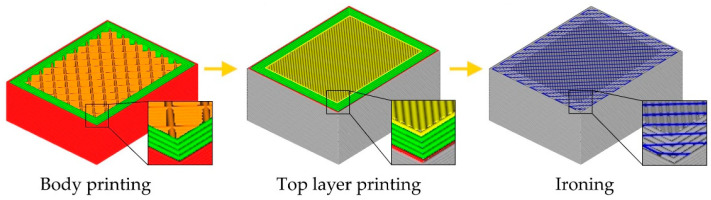
Stages of performed research.

The ironing pattern was set to zig-zag. The top surface ironing process, due to the pressure of the nozzle on the ironed surface, results in a greater load on the printer. Due to the need to ensure stable printer operation, the acceleration has been set to 1000 mm/s^2^. The ironing process parameters were selected based on literature analysis and own experience. The impact of the ironing process on roughness values (Ra_p_) and flatness (FLTq_p_) was determined. The diagram illustrating the used research methodology is shown in Figure 1.

### 2.2. Performing Experimental Research

Samples with base dimensions of 20 mm × 25 mm and height of 10 mm were used to conduct the tests. The sample model was designed in CAD Fusion 360 software (Autodesk, San Rafael, CA, USA). UltiMaker Cura 5.6.0 (Ultimaker B.V., New York, NY, USA) was used as a slicer program. G-codes used during the post-processing process were additionally edited manually after their generation. This action was necessary due to the lack of functionality in the slicer used to determine the extruder temperature value used during the ironing process.

The samples were made with FDM 3D printer Creality Ender 3 V3 SE (Shenzhen Creality 3D Technology, Shenzhen, China) with a Direct Drive extruder. In this study Creality Ender PLA+ filament was used. Each set of tested parameters was repeated three times. Samples were made in random order.

**Figure 2:**
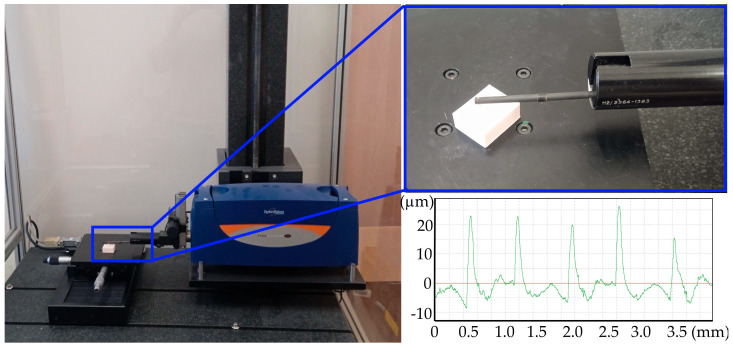
The test stand used for measurements and an example view of the roughness profile of the measured sample.

Surface roughness and flatness measurements were performed using a Form TalySurf Intra 50 profilographometer (Taylor Hobson, Leicester, UK). Surface roughness analysis was performed based on the ISO 4287 standard [26] for l_c_ = 0.8 mm. Flatness measurements were performed with a 0.5 mm margin on the entire surface of the sample with a resolution of 241 × 19 points in the x and y axes. A diamond cone-shaped measuring tip with a rounding radius of 2 μm was used to measure surface roughness. A ruby ball-shaped tip with a radius of 1 mm was used to measure flatness. Surface roughness was prepared three times on each sample, while flatness was measured once. As a result of the measurements, 378 data points were obtained for surface roughness and 126 for surface flatness. Figure 2 shows a view of the test stand used for measurements and an example view of the roughness profile of the measured sample.

Microscopic images of the obtained surfaces were also analyzed. A digital microscope Keyonce VHX-600 (Keyence, Osaka, Japan) with automatic *Z*-axis drive and a lens with 50 and 200 times magnification was used.

### 2.3. Application of the Response Surface Methodology in Process Modeling

The Response Surface Methodology (RSM) and the central composition plan were used to create mathematical models and analyze and optimize the process parameters. The analyses were performed in Statistica software (13, TIBCO Software., San Ramon, CA, USA) for the assumed significance level of α = 0.05. The types of plan points and coded and set values of the tested parameters used during the first stage (analysis and optimization of the top surface printing process) are presented in Table 3.

Table 4 summarizes the types of plan points and coded and set values of the tested parameters used during the second stage (analysis and optimization of the top surface ironing process).

Mathematical models were created based on a second-order polynomial regression equation with interactions between factors. This model can be written as follows (1).

(1) $$Y=\beta_{0}+\sum_{i=1}^{k}\beta_{i}x_{i}+\sum_{i=1}^{k}\beta_{ii}x_{i}^{2}+\sum_{i<j}^{k}\beta_{ij}x_{i}x_{j}\pm \varepsilon ,$$

where *k*—number of input process parameters, *x_i_*—input variables, *x_i_*^2^ and *x_i_x_j_*—the squares and interaction terms, respectively, *β*_0_—constant, *β_i_*, *β_ii_*, *β_ij_*—coefficients of linear, quadratic, and cross product terms, respectively, and *e*—error.

The evaluation of the created models was based on the calculation of Determination Coefficients *R* (2) and their squares *R*^2^, Relative Absolute Deviations *RAD* (3), and Average Deviations *AD* (4).

(2) $$R=\frac{\sum_{i=1}^{N}\left(E_{i}-\bar{E}\right)\cdot \left(O_{i}-\bar{O}\right)}{\sqrt{\sum_{i=1}^{N}{\left(E_{i}-\bar{E}\right)}^{2}}\cdot \sqrt{\sum_{i=1}^{N}{\left(O_{i}-\bar{O}\right)}^{2}}}$$

(3) $$RAD=\frac{\sum_{i=1}^{N}\left|E_{i}-O_{i}\right|/E_{i}}{N-1}$$

(4) $$AD=\frac{\sum_{i=1}^{N}\left|E_{i}-O_{i}\right|}{N-1}$$

where *N*—number of observations; *E_i_*—predicted value of case i; $\bar{E}$—mean of predicted values; *O_i_*—observed value of case *I*; and $\bar{O}$—mean of observed values.

## 3. Results and Discussion

### 3.1. Modeling of Top Surface Printing Process

**Figure 3:**
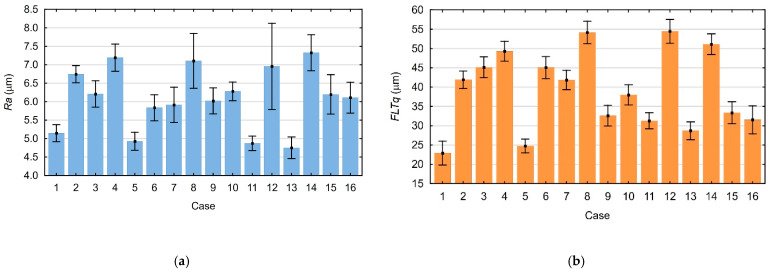
Mean values and standard deviations of parameters measured on the printed top surfaces: (**a**) Ra parameter; (**b**) FLTq parameter.

In order to create a mathematical model describing the top surface printing process, the data obtained during the measurements were analyzed. Figure 3a,b show the measured average values of the Ra and FLTq parameters with their standard deviations.

**Figure 4:**
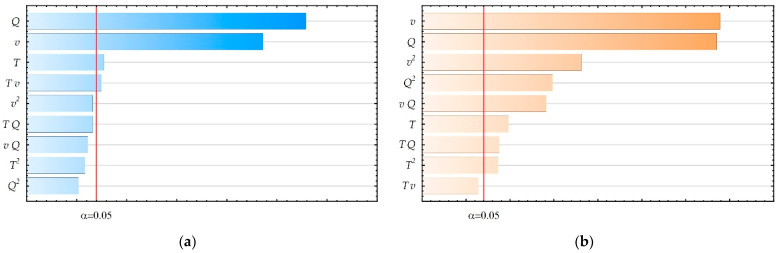
Pareto diagrams: (**a**) Ra parameter; (**b**) FLTq parameter.

The impact of the tested input parameters on the output parameter values was also assessed. For this purpose, ANOVA (Appendix A, Table A1 and Table A2) and Pareto diagram (Figure 4a,b) were used. The results of both analyses are convergent.

**Figure 5:**
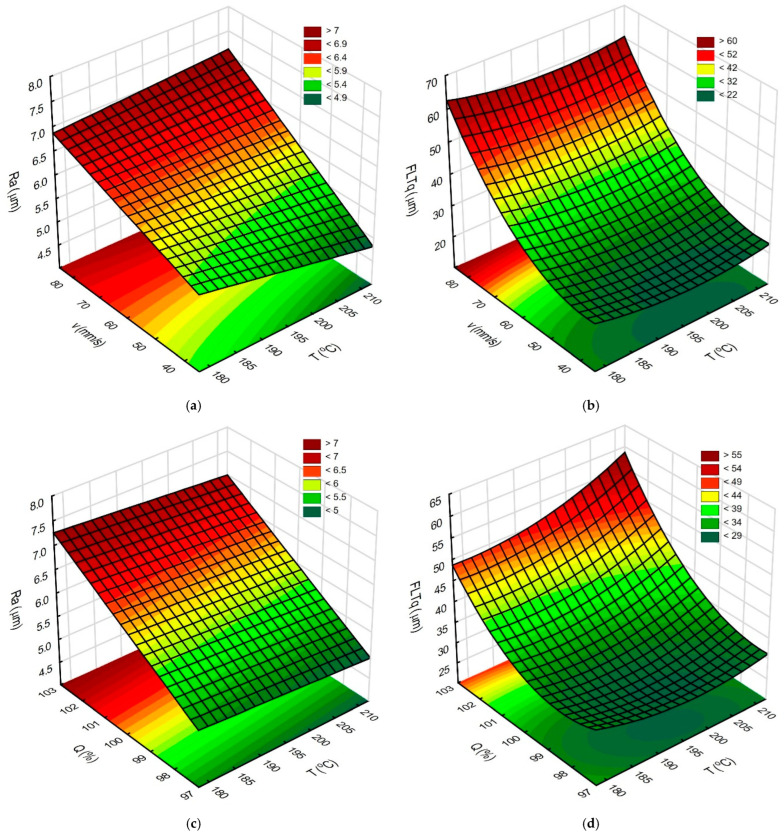
The response surfaces: (**a**) influence of T and v on Ra parameter, constant Q = 100%; (**b**) influence of T and v on FLTq parameter, constant Q = 100%; (**c**) influence of T and Q on Ra parameter, constant v = 60 mm/s; and (**d**) influence of T and Q on FLTq parameter, constant v = 60 mm/s.

In the next step, the response surfaces were determined. They were created without input parameters whose influence on the values of output parameters was not proven by ANOVA and Pareto analysis. The response surfaces are shown in Figure 5a–d.

The created models were also evaluated, and the analysis results are presented in Table 5.

Based on the measurements, differences in the Ra parameter values are observed both in different areas of one surface and on different surfaces made within repetitions. A number of repeatable surfaces were obtained, characterized by average repeatability of the geometric structure. In the case of measurements of the FLTq parameter, lower values of standard deviations were obtained, which indicates greater repeatability.

The strongest influence on the Ra parameter is exerted by filament flow and printing speed, which are linear in both cases. The linear influence of extruder temperature and nonlinear influence resulting from the interaction of extruder temperature and printing speed were also observed on the Ra parameter values. Lack of influence on the Ra parameter value was demonstrated for the remaining tested interactions. Analysis of the impact of input parameters on the FLTq parameter values also showed a strong linear effect of printing speed and filament flow. In addition to the interaction between extruder temperature and printing speed, the influence of all other tested input parameters or their interactions was demonstrated. Additionally, the Pearson correlation coefficient was calculated for the measured values of Ra and FLTq parameters. The value of this coefficient is equal to r = 0.869, which means that there is a strong correlation between the obtained surface roughness and flatness.

Analyzing the response surfaces (Figure 5), a negative effect of printing speed and increasing filament flow during top surface printing is noticeable. Using high printing speed and filament flow values simultaneously, a deterioration in the flatness of the produced surfaces is observed. Using higher extruder temperatures results in obtaining surfaces with lower roughness. Setting the middle-tested extruder temperature value improves the flatness of the obtained surfaces.

### 3.2. Optimization of Top Surface Printing Process

**Figure 6:**
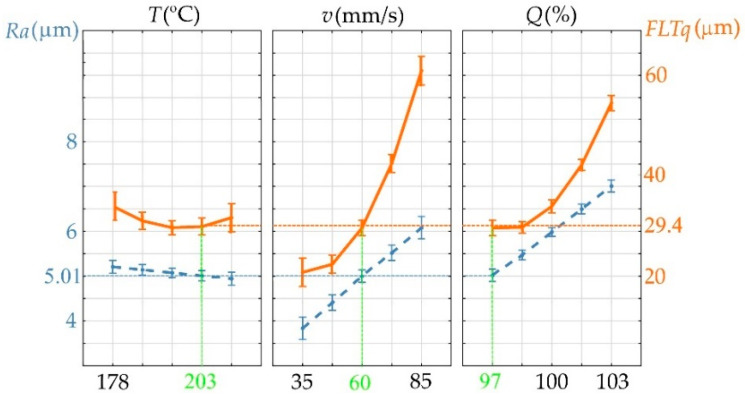
Optimization graph for Ra and FLTq parameters.

The aim of top surface 3D printing process optimization was to minimize the surface roughness parameter Ra and flatness parameter FLTq. The optimization process was performed based on the previously created mathematical model (Section 3.1). To ensure high process efficiency, the selection of printing parameters was optimized for the average printing speed used (v = 60 mm/s). During the optimization process, the filament flow rate and extruder temperature were selected. The optimization graph is shown in Figure 6.

**Figure 7:**
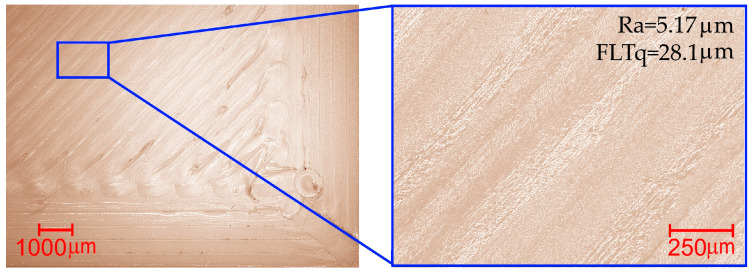
Microscopic images of the upper surface of the sample made using optimal parameters (T = 203 °C, v = 60 mm/s, and Q = 97%).

It was determined that the optimal top surface printing parameters would be T = 203 °C and Q = 97%. For the optimal values of the input parameters, Ra = 5.01 ± 0.65 μm and FLTq = 29.4 ± 3.5 μm were calculated. To verify the optimization process, three samples were printed and Ra and FLTq were measured. The measured values are consistent with those calculated during the optimization process, and their averages are Ra = 5.17 μm with a std. dev. of 0.21 and FLTq = 28.1 μm with a std. dev. of 1.12. The surface obtained as a result of the optimization process is still characterized by defects. Microscopic images of the top surface of the sample made using the optimal printing parameters are shown in Figure 7.

### 3.3. Modeling of Top Surface Ironing Process

To determine the effect of the analyzed ironing process parameters on the top surface roughness (Ra_p_) and flatness (FTLq_p_), samples were prepared. In the first step, the top surface of the sample was printed using optimal printing parameters: T = 203 °C, v = 60 mm/s, and Q = 97% (Section 3.2). Then, immediately after top surface printing, the sample was ironed. Ironing processes were made based on the conditions listed in Table 4. During the extruder warm-up stage (between the end of top surface printing and the start of ironing), it was noticed that a small amount of filament flows out from the extruder. This phenomenon can interfere with the surface ironing process. Therefore, automatic cleaning of the extruder nozzle was performed immediately before ironing.

**Figure 8:**
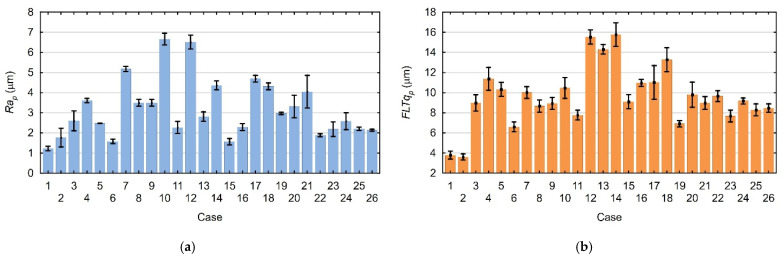
Mean values of measured parameters with their standard deviations: (**a**) Ra_p_ parameter; (**b**) FLTq_p_ parameter.

Figure 8a,b show the measured average values of the Ra_p_ and FLTq_p_ parameters and their standard deviations.

**Figure 9:**
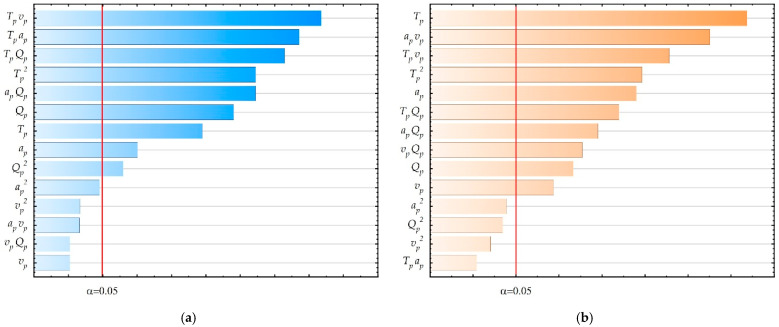
Pareto diagrams for: (**a**) Ra_p_ parameter; (**b**) FLTq_p_ parameter.

The influence of the significance of tested input parameters on the values of output parameters was assessed using ANOVA and Pareto diagrams. The ANOVA tables are placed in Appendix A, Table A3 and Table A4. Pareto diagrams are provided in Figure 9a,b. The results of both analyses are convergent.

**Figure 10:**
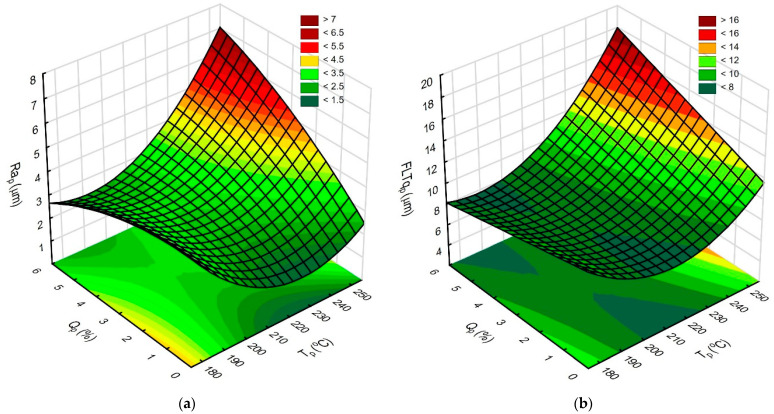
The response surfaces illustrating the influence of T_p_ and Q_p_ on (**a**) Ra_p_ parameter, constant v_p_ = 50 mm/s, and a_p_ = 0.3 mm; (**b**) FLTq_p_ parameter, constant v_p_ = 50 mm/s, and a_p_ = 0.3 mm.

The next step involved determining the response surfaces. These models were created by omitting the input variables for which ANOVA and Pareto analysis did not prove their influence on the Ra_p_ and FLTq_p_ parameter values. The response surfaces illustrating the effect of extruder temperature T_p_ and filament flow Q_p_ are shown in Figure 10a,b.

**Figure 11:**
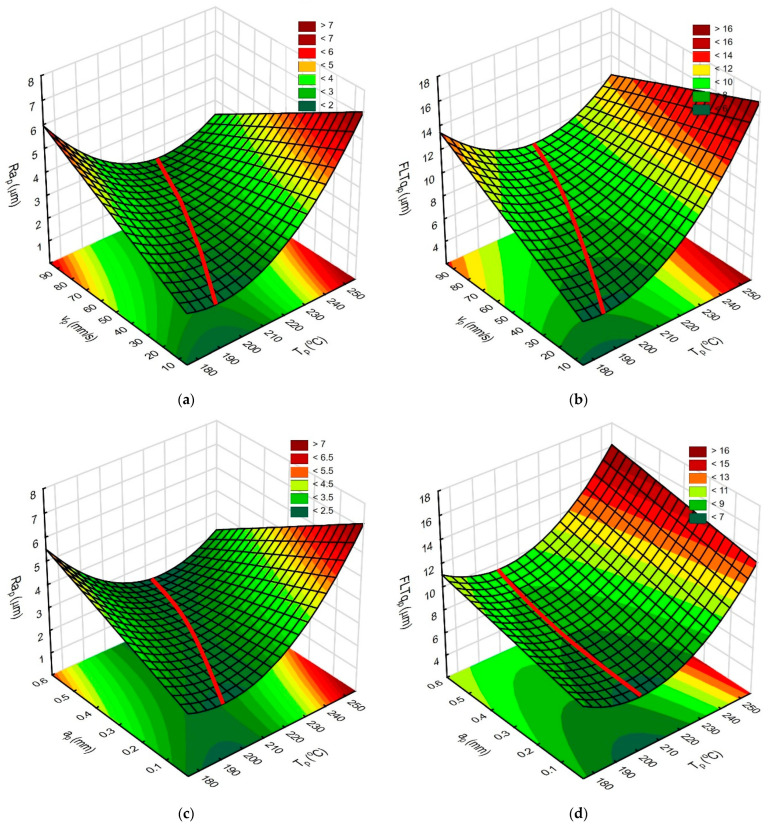
The response surfaces illustrating the impact of T_p_, v_p_, and a_p_: (**a**) influence of T_p_ and v_p_ on Ra_p_ parameter, constant a_p_ = 0.3 mm and Q = 3%; (**b**) influence of T_p_ and v_p_ on FLTq_p_ parameter, constant a_p_ = 0.3 mm and Q_p_ = 3%; (**c**) influence of T_p_ and a_p_ on Ra_p_ parameter, constant v_p_ = 50 mm/s and Q = 3%; (**d**) influence of T_p_ and a_p_ on FLTq_p_ parameter, constant v_p_ = 50 mm/s and Q = 3%.

Figure 11a−d show graphs illustrating the influence of T_p_, v_p_, and a_p_ on surface roughness and flatness. The red line indicates the relationship between the analyzed parameters, ensuring the achievement of minimum roughness and flatness values.

The created models were also evaluated and the analysis results are presented in Table 6.

The performed ANOVA and Pareto analyses showed a strong nonlinear influence of most of the tested input parameters or interactions of input parameters. The greatest influence on surface roughness is exerted by changes in extruder temperature, although it is also observed in interaction with other tested ironing process parameters. A strong influence on surface roughness was also observed for a_p_ and v_p_ parameters. It was noticed that similar dependencies were obtained for the parameter FLTq_p_. Additionally, the simultaneous influence of changes in v_p_ and a_p_ and the nonlinear influence of v_p_ were noticed. The Pearson correlation coefficient for the values of the Ra_p_ and FLTq_p_ parameters is r = 0.62, which means a weak correlation between the obtained surface roughness and flatness.

Based on the created response surfaces (Figure 10), small changes in Ra_p_ and FLT_p_ were observed when using the lowest tested T_p_ and varied Q_p_ values. Increasing T_p_ to 215 °C improves both surface roughness and flatness. Above this T_p_ range, the adverse effect of increasing T_p_ and Q_p_ is visible. Studying the above graphs shows that, for the analyzed ironing process, filament extrusion during the ironing process should be abandoned. This approach allows the filament to be withdrawn from the extruder nozzle. This means that, during a possible heating of the extruder to the ironing temperature, the filament will not flow out freely and the nozzle cleaning procedure can be abandoned.

In Figure 11, the varied influence of v_p_ and a_p_ on the state of the obtained surface is visible. For the lowest tested T_p_, a negative influence of increasing v_p_ and a_p_ is observed. This state is maintained up to the extruder temperature of about 215 °C. Above this extruder temperature, a beneficial influence of increasing v_p_ and a_p_ on Ra_p_ and v_p_ on FLTq_p_ is observed, while the unfavorable influence of a_p_ on FLTq_p_ is still visible.

**Figure 12:**
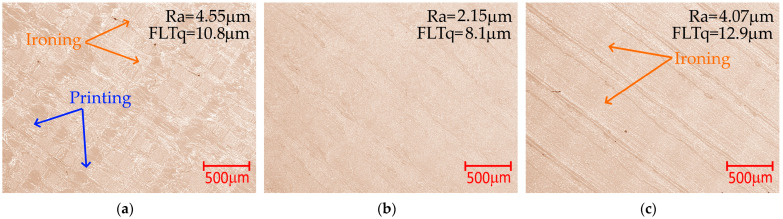
Microscopic photos of surfaces made using different extruder temperatures: (**a**) Case 17, T_p_ = 178 °C; (**b**) Case 25, T_p_ = 215 °C; (**c**) Case 18, T_p_ = 252 °C; constant parameter a_p_ = 0.3 mm, v_p_ = 50 mm/s, Q_p_ = 3%.

Simultaneous use of the lowest tested T_p_ and the highest v_p_ and a_p_ or the use of T_p_ > 240 °C and low v_p_ and a_p_ values causes deterioration of the surface roughness (Figure 11a, c; Ra_p_ > 4.96 μm). The use of the lowest tested T_p_ does not undergo the filament flow process, while the nozzle pressure on the ironed surface causes mechanical damage. When using the highest tested T_p_ values, the filament is excessively heated and flows out under the nozzle, which causes grooves and they are observed on the surface of the sample. Microscopic photos of surfaces made using different extruder temperatures are shown in Figure 12a−c.

### 3.4. Optimization of Top Surface Ironing Process

Based on the analysis of the mathematical models describing the top surface ironing process developed in Section 3.3, the optimization criteria were selected and the extruder temperature values used during ironing were determined. The goal of optimization was to minimize surface roughness and increase flatness. Zero filament flow was applied during the ironing process. The temperature was determined for three different sets of v_p_ and a_p_, representing three levels of ironing process efficiency. Table 7 presents the assumed ironing parameters v_p_, a_p_, Q_p_, and the predicted ironing temperature. The Ra_p_ and FLTq_p_ values obtained from the developed models (presented in Section 3.3) as well as those measured on the samples after ironing are also presented in Table 7. The optimal parameters (T = 203 °C, v = 60 mm/s, and Q = 97%) established in Section 3.2 were used to print the top surface of the samples.

By analyzing Table 7 for v_p_ = 20 mm/s and a_p_ = 0.1 mm, the Ra_p_ values calculated using the developed models are underestimated in relation to the measured values. The RSM used approximates the solution space with a second-degree polynomial, which introduces some generalization into the model. The model takes into account only the analyzed process parameters, while complex material flow phenomena affect the result. It is also known that polynomial models work best near the center of the plan. Outside this region, less accurate results may be obtained. The observed results may also indicate that the process has reached its technological limit for the used test stamp. In order to confirm this hypothesis, several additional samples were produced at speeds lower than 20 mm/s and with distances between passes smaller than 0.1 mm. As a result of these tests, no further improvement in the surface roughness of the samples was observed. The results continued to oscillate around Ra = 1 μm. Regardless, the surfaces obtained are characterized by high quality. The application of the analyzed two-stage method yields better effects than those reported by other researchers using the ironing process. Butt et al. [21] and Sardinha et al. [22] report surface roughness values of approximately Ra = 9 µm on ABS samples at ironing efficiencies of 10 mm^2^/s and 2 mm^2^/s, respectively. Caputo et al. [23] obtain a significantly lower roughness of Ra = 0.82 μm; however, they perform time-consuming ironing of each layer, and the used ironing efficiency is 1.6 mm^2^/s. In turn, the authors [24] select a set of parameters that allows Ra = 2.73 μm to be obtained at ironing efficiency of 3.2 mm^2^/s on a PLA sample. However, they simultaneously optimize the surface roughness and mechanical performance of the samples.

**Figure 13:**
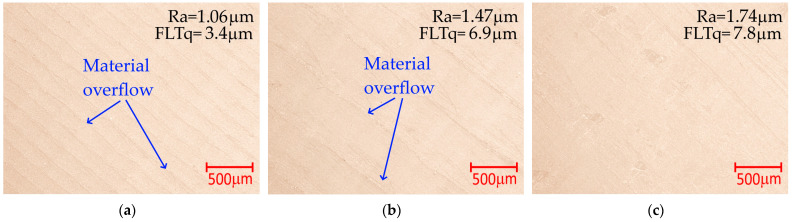
Microscopic photos of surfaces made using different ironing parameters: (**a**) T_p_ = 198 °C, a_p_ = 0.1 mm and v_p_ = 20 mm/s; (**b**) T_p_ = 225 °C, a_p_ = 0.3 mm and v_p_ = 50 mm/s; (**c**) T_p_ = 245 °C, a_p_ = 0.5 mm and v_p_ = 80 mm/s; constant parameter Q_p_ = 0%.

During the analyses, microscopic images of the surfaces obtained as a result of ironing were also taken. The images indicate marks left by the printer extruder nozzle and material overflow. Their width increases with increasing speed and distance between passages to values of v_p_ = 50 mm/s and a_p_ = 0.3 mm, respectively. This results in increased roughness. At a speed of v_p_ = 80 mm/s and a distance between passages of a_p_ = 0.5 mm, they are no longer clearly visible. The outflow is also observed on surfaces fabricated by other researchers [22,23,24]. Figure 13 shows microscopic views of the top surfaces of samples ironed using the parameter sets from Table 7.

## 4. Conclusions

This study focused on modeling the process of ironing the upper surface of the manufactured element. Ironing the upper surface is one of the possible post-processing methods that allow, among others, improving the quality of the obtained surface. The RSM method was used to model the printing processes using MEX technology and ironing the surface as experimental studies were performed. To assess the produced surfaces, the values of surface roughness parameter Ra and surface flatness FLTq were analyzed. The proposed research methodology includes two main stages. In the first stage, the influence of printing speed, extruder temperature, and filament flow on the quality of the obtained surface was determined. The values of extruder temperature and filament flow were optimized. The optimization criterion was to minimize surface roughness and flatness while maintaining high process efficiency. After the first stage optimization, a set of parameters (T = 203 °C and Q = 97%) was selected to allow for the production of a surface characterized by Ra = 5.17 μm and FLTq = 28.1 μm with a printing speed of 60 mm/s. In the second stage, the influence of extruder temperature, ironing speed, distance between passes, and filament flow used during the ironing process was modeled. Graphs were created to select the values of the analyzed ironing parameters that ensure obtaining surfaces characterized by high quality. As a result of the tests and analyses performed, the following main conclusions were formulated:

1. A strong influence of printing speed and filament flow on the roughness and flatness of surfaces produced using MEX technology was investigated.

2. As a result of tests, the interactions between the extruder temperature used during the surface ironing process and the ironing speed, the distance between the passes, and the filament flow were observed. Increasing the ironing speed and the distance between the passes should simultaneously increase the extruder temperature.

3. It is possible to select a constant extruder temperature value used in the 3D printing and ironing process due to the high quality of the obtained surfaces and ensuring high process efficiency. There is no need for additional heating of the extruder, during which the phenomenon of free filament flow was observed.

4. As a result of the tests and analyses, it was shown that it is reasonable to abandon the additional filament flow used during the ironing process. Such action allows for the complete withdrawal of the filament from the extruder nozzle, which prevents the phenomenon of free filament flow.

5. Surface ironing, which was carried out based on correctly selected process parameters, resulted in a fivefold decrease in the Ra parameter value and an eightfold decrease in the FLTq parameter value (Ra_p_ = 1.09 μm and FLTq_p_ = 3.4 μm). The consequence of incorrect selection of ironing process parameters may be the observed deterioration of surface roughness.

In addition to summarizing the key findings, several limitations of this study should be noted. Mathematical models developed using RSM are based on polynomial regression equations, which provide a locally correct description of the process in the experimental space. The experimental modeling used does not fully describe the physical phenomena occurring and is susceptible to the characteristics of the test setup used. As a result, the created models do not fully reflect the phenomena related to the complex material flow during the ironing process. RSM also has limited capabilities for performing multi-criteria and multi-parametric optimizations. The two-stage method is more complex, requiring two separate experiments and a larger number of trials and measurements. Optimizing each stage separately may omit combinations that would yield a better final result. Furthermore, it is not precisely known what surface parameters obtained from printing the top layer are required to achieve the desired ironing results. The ironing process is an additional step. Its implementation and the additional heating of the extruder nozzle extend the time required to obtain the product. During the nozzle heating procedure, free flow of material from the extruder was observed. If a non-zero filament flow rate is used during ironing, a nozzle cleaning procedure is necessary. Otherwise, the produced surfaces are characterized by artifacts. Current CAM software used to program 3D printers do not include the functionality to set different extruder temperatures for printing and ironing, requiring manual modification of the code. Despite these limitations, the results obtained in this study have both scientific and practical value. From a scientific perspective, the work provides a statistically supported source for systematic assessments of the effect of ironing efficiency and temperature on surface roughness and flatness. The described findings deepen our understanding of the mechanisms responsible for material redistribution during ironing and highlight the interrelationships between the analyzed parameters. From a practical perspective, the proposed two-stage ironing strategy enables the production of surfaces with significantly lower roughness levels than those reported in previous studies, while maintaining process efficiency and eliminating the need for additional equipment or substances.

In summary the above observations and recommendations and the application of the research methodology proposed in this study enables simultaneous selection of the 3D printing parameters and surface ironing processes. This makes it possible to efficiently produce aesthetic surfaces characterized by low roughness and flatness values.

## Figures and Tables

**Table 1:** The tested top surface printing parameters levels.

Parameter	Values
Extruder temperature, T (°C)	178; 185; 195; 205; 212
Printing speed, v (mm/s)	34.8; 45; 60; 75; 85.2
Filament flow, Q (%)	96.64; 98; 100; 102; 103.36

**Table 2:** The tested top surface ironing parameters levels.

Parameter	Values
Extruder temperature, T_p_ (°C )	178; 190; 215; 240; 252
Ironing speed, v_p_ (mm/s)	5.5; 20; 50; 80; 94.5
Passes distance, a_p_ (mm)	0.01; 0.1; 0.3; 0.5; 0.59
Filament flow, Q_p_ (%)	0.03; 1; 3; 5; 5.97

**Table 3:** The types of plan points and coded and set values of the tested parameters used during the printing optimization stage.

Case	Point Type	*A*	*B*	*C*	*T* (°C)	*v* (mm/s)	*Q* (%)
1	factorial	−1	−1	−1	185	45	98
2	factorial	−1	−1	1	185	45	102
3	factorial	−1	1	−1	185	75	98
4	factorial	−1	1	1	185	75	102
5	factorial	1	−1	−1	205	45	98
6	factorial	1	−1	1	205	45	102
7	factorial	1	1	−1	205	75	98
8	factorial	1	1	1	205	75	102
9	axial	−1.682	0	0	178	60	100
10	axial	1.682	0	0	212	60	100
11	axial	0	−1.682	0	195	34.8	100
12	axial	0	1.682	0	195	85.2	100
13	axial	0	0	−1.682	195	60	96.64
14	axial	0	0	1.682	195	60	103.36
15	center	0	0	0	195	60	100
16	center	0	0	0	195	60	100

**Table 4:** The types of plan points and coded and set values of the tested parameters used during the ironing optimization stage.

Case	Point Type	*A*	*B*	*C*	*D*	*T_p_* (°C)	*a_p_* (mm)	*v_p_* (mm/s)	*Q_p_* (%)
1	factorial	−1	−1	−1	−1	190	0.1	20	1
2	factorial	−1	−1	−1	1	190	0.1	20	5
3	factorial	−1	−1	1	−1	190	0.1	80	1
4	factorial	−1	−1	1	1	190	0.1	80	5
5	factorial	−1	1	−1	−1	190	0.5	20	1
6	factorial	−1	1	−1	1	190	0.5	20	5
7	factorial	−1	1	1	−1	190	0.5	80	1
8	factorial	−1	1	1	1	190	0.5	80	5
9	factorial	1	−1	−1	−1	240	0.1	20	1
10	factorial	1	−1	−1	1	240	0.1	20	5
11	factorial	1	−1	1	−1	240	0.1	80	1
12	factorial	1	−1	1	1	240	0.1	80	5
13	factorial	1	1	−1	−1	240	0.5	20	1
14	factorial	1	1	−1	1	240	0.5	20	5
15	factorial	1	1	1	−1	240	0.5	80	1
16	factorial	1	1	1	1	240	0.5	80	5
17	axial	−1.483	0	0	0	178	0.3	50	3
18	axial	1.483	0	0	0	252	0.3	50	3
19	axial	0	−1.483	0	0	215	0.01	50	3
20	axial	0	1.483	0	0	215	0.59	50	3
21	axial	0	0	−1.483	0	215	0.3	5.5	3
22	axial	0	0	1.483	0	215	0.3	94.5	3
23	axial	0	0	0	−1.483	215	0.3	50	0.03
24	axial	0	0	0	1.483	215	0.3	50	5.97
25	center	0	0	0	0	215	0.3	50	3
26	center	0	0	0	0	215	0.3	50	3

**Table 5:** Evaluation results of the created models.

Source	*R*	*R* ^2^	*RAD*	*AD*
*Ra*	0.923	0.861	0.044	0.265
*FLTq*	0.990	0.981	0.031	1.142

**Table 6:** Evaluation results of the created models.

Source	*R*	*R* ^2^	*RAD*	*AD*
*Ra*_p_	0.902	0.813	0.163	0.472
*FLTq*_p_	0.950	0.902	0.073	0.690

**Table 7:** Sets of ironing parameters with predicted and measured surface roughness and flatness values.

Ironing Parameter				Predicted Value (95% CI)		Measured (Min–Max)		Measured (Average)		Ironing Efficiency
*T_p_* (°C )	*v_p_* (mm)	*a_p_* (mm/s)	*Q_p_* (%)	Ra_p_ (μm)	FLTq_p_ (μm)	Ra_p_ (μm)	FLTq_p_ (μm)	Ra_p_ (μm)	FLTq_p_ (μm)	E_p_ (mm^2^/s)
198	20	0.1	0	0.32–1.04	3.1–5.0	0.99–1.23 *	2.8–4.1	1.09 *	3.4	2
225	50	0.3	0	1.19–1.74	7.5–8.7	1.32–1.69	7.0–8.3	1.51	7.6	15
245	80	0.5	0	1.06–2.34	7.1–9.4	1.14–1.85	7.1–8.5	1.57	7.8	40

* indicates a value out of range.

## Data Availability

The original contributions presented in this study are included in the article. Further inquiries can be directed to the corresponding author.

## Appendix A

**Table A1:** The ANOVA results for Ra.

Source	Seq SS	DF	MS	F-Value	*p*-Value
**T**	0.75	1	0.75	7.49	0.007
**T^2^**	0.07	1	0.07	0.69	0.408 *
**v**	34.79	1	34.79	346.76	0.000
**v^2^**	0.27	1	0.27	2.65	0.106 *
**Q**	52.54	1	52.54	523.77	0.000
**Q^2^**	0.003	1	0.003	0.03	0.860 *
**T ^.^ v**	0.61	1	0.61	6.13	0.015
**T ^.^ Q**	0.26	1	0.26	2.61	0.109 *
**v ^.^ Q**	0.12	1	0.12	1.24	0.268 *
**Residual Error**	13.44	134	0.10		
**Total**	103.05	143			

Seq SS—the sum of squares due to the source. DF—the degrees of freedom in the source. MS—the mean sum of squares due to the source. F-Value—the variance of the group means. *p*-Value—the probability that the difference between groups is due to chance. The * indicates non important factors with a statistically non-significant influence on the measurement results.

**Table A2:** The ANOVA results for FLTq.

Source	Seq SS	DF	MS	F-Value	*p*-Value
**T**	54.56	1	54.56	23.14	0.000
**T^2^**	31.13	1	31.13	13.20	0.001
**v**	1970.24	1	1970.24	835.71	0.000
**v^2^**	404.64	1	404.64	171.64	0.000
**Q**	1916.45	1	1916.45	812.90	0.000
**Q^2^**	228.52	1	228.52	96.93	0.000
**T ^.^ v**	4.54	1	4.54	1.93	0.173 *
**T ^.^ Q**	33.56	1	33.56	14.23	0.001
**v ^.^ Q**	195.97	1	195.97	83.12	0.000
**Residual Error**	89.59	38	2.36		
**Total**	4762.75	47			

Seq SS—the sum of squares due to the source. DF—the degrees of freedom in the source. MS—the mean sum of squares due to the source. F-Value—the variance of the group means. *p*-Value—the probability that the difference between groups is due to chance. The * indicates non important factors with a statistically non-significant influence on the measurement results.

**Table A3:** The ANOVA results for Ra_p_.

Source	Seq SS	DF	MS	F-Value	*p*-Value
**T_p_**	24.73	1	24.73	60.66	0.000
**T_p_^2^**	48.40	1	48.40	118.71	0.000
**a_p_**	6.60	1	6.60	16.19	0.000
**a_p_^2^**	1.34	1	1.34	3.28	0.071 *
**v_p_**	0.003	1	0.003	0.01	0.934 *
**v_p_^2^**	0.18	1	0.18	0.44	0.506 *
**Q_p_**	37.72	1	37.72	92.51	0.000
**Q_p_^2^**	4.17	1	4.17	10.22	0.002
**T_p_^.^ a_p_**	73.59	1	73.59	180.51	0.000
**T_p_^.^ v_p_**	88.34	1	88.34	216.68	0.000
**T_p_^.^ Q_p_**	64.46	1	64.46	158.11	0.000
**a_p_^.^ v_p_**	0.17	1	0.17	0.41	0.523 *
**a_p_^.^ Q_p_**	48.32	1	48.32	118.53	0.000
**v_p_^.^ Q_p_**	0.003	1	0.003	0.01	0.927 *
**Residual Error**	89.28	219	0.41		
**Total**	487.31	233			

Seq SS—the sum of squares due to the source. DF—the degrees of freedom in the source. MS—the mean sum of squares due to the source. F-Value—the variance of the group means. *p*-Value—the probability that the difference between groups is due to chance. The * indicates non important factors with a statistically non-significant influence on the measurement results.

**Table A4:** The ANOVA results for FLTq_p_.

Source	Seq SS	DF	MS	F-Value	*p*-Value
**T_p_**	158.22	1	158.22	162.24	0.000
**T_p_^2^**	60.21	1	60.21	61.74	0.000
**a_p_**	56.32	1	56.32	57.75	0.000
**a_p_^2^**	2.32	1	2.32	2.38	0.128 *
**v_p_**	13.61	1	13.61	13.96	0.000
**v_p_^2^**	0.66	1	0.66	0.68	0.414 *
**Q_p_**	21.13	1	21.13	21.67	0.000
**Q_p_^2^**	1.85	1	1.85	1.90	0.173 *
**T_p_^.^ a_p_**	0.03	1	0.03	0.03	0.868 *
**T_p_^.^ v_p_**	81.15	1	81.15	83.21	0.000
**T_p_^.^ Q_p_**	44.92	1	44.92	46.06	0.000
**a_p_^.^ v_p_**	118.10	1	118.10	121.10	0.000
**a_p_^.^ Q_p_**	32.93	1	32.93	33.77	0.000
**v_p_^.^ Q_p_**	25.18	1	25.18	25.82	0.000
**Residual Error**	61.44	63	0.98		
**Total**	678.08	77			

Seq SS—the sum of squares due to the source. DF—the degrees of freedom in the source. MS—the mean sum of squares due to the source. F-Value—the variance of the group means. *p*-Value—the probability that the difference between groups is due to chance. The * indicates non important factors with a statistically non-significant influence on the measurement results.
